# “I am feeling tension in my whole body”: An experimental phenomenological study of empathy for pain

**DOI:** 10.3389/fpsyg.2022.999227

**Published:** 2023-01-04

**Authors:** David Martínez-Pernía, Ignacio Cea, Alejandro Troncoso, Kevin Blanco, Jorge Calderón Vergara, Constanza Baquedano, Claudio Araya-Veliz, Ana Useros-Olmo, David Huepe, Valentina Carrera, Victoria Mack Silva, Mayte Vergara

**Affiliations:** ^1^School of Psychology, Center for Social and Cognitive Neuroscience (CSCN), Universidad Adolfo Ibáñez, Santiago, Chile; ^2^Faculty of Medicine, Geroscience Center for Brain Health and Metabolism (GERO), University of Chile, Santiago, Chile; ^3^Faculty of Medicine, Memory and Neuropsychiatric Clinic (CMYN), Neurology Service, Hospital del Salvador, University of Chile, Santiago, Chile; ^4^Philosophy Department, Faculty of Philosophy and Humanities, Universidad Alberto Hurtado, Santiago, Chile; ^5^School of Psychology, Universidad Adolfo Ibáñez, Santiago, Chile; ^6^Unidad de Daño Cerebral, Hospital Beata María Ana, Madrid, Spain; ^7^Departamento de Fisioterapia and Motion in Brains Research Group, Institute of Neuroscience and Sciences of the Movement (INCIMOV), Centro Superior de Estudios Universitarios La Salle, Universidad Autónoma de Madrid, Madrid, Spain

**Keywords:** empathy for pain, experimental phenomenology, neurophenomenology, enaction, first-person view, bodily sensation, social emotion, extreme sport

## Abstract

**Introduction:**

Traditionally, empathy has been studied from two main perspectives: the theory-theory approach and the simulation theory approach. These theories claim that social emotions are fundamentally constituted by mind states in the brain. In contrast, classical phenomenology and recent research based on the enactive theories consider empathy as the basic process of contacting others’ emotional experiences through direct bodily perception and sensation.

**Objective:**

This study aims to enrich the knowledge of the empathic experience of pain using an experimental phenomenological method.

**Materials and methods:**

Implementing an experimental paradigm used in affective neuroscience, we exposed 28 healthy adults to a video of sportspersons suffering physical accidents while practicing extreme sports. Immediately after watching the video, each participant underwent a phenomenological interview to gather data on embodied, multi-layered dimensions (bodily sensations, emotions, and motivations) and temporal aspects of empathic experience. We also performed quantitative analyses of the phenomenological categories.

**Results:**

Experiential access to the other person’s painful experience involves four main themes. Bodily resonance: participants felt a multiplicity of bodily, affective, and kinesthetic sensations in coordination with the sportsperson’s bodily actions. Attentional focus: some participants centered their attention more on their own personal discomfort and sensations of rejection, while others on the pain and suffering experienced by the sportspersons. Kinesthetic motivation: some participants experienced the feeling in their bodies to avoid or escape from watching the video, while others experienced the need to help the sportspersons avoid suffering any injury while practicing extreme sports. The temporality of experience: participants witnessed temporal fluctuations in their experiences, bringing intensity changes in their bodily resonance, attentional focus, and kinesthetic motivation. Finally, two experiential structures were found: one structure is self-centered empathic experience, characterized by bodily resonance, attentional focus centered on the participant’s own experience of seeing the sportsperson suffering, and self-protective kinesthetic motivation; the other structure is other-centered empathic experience, characterized by bodily resonance, attentional focus centered on the sportsperson, and prosocial kinesthetic motivation to help them.

**Discussion:**

We show how phenomenological data may contribute to comprehending empathy for pain in social neuroscience. In addition, we address the phenomenological aspect of the enactive approach to the three dimensions of an embodiment of human consciousness, especially the intersubjective dimension. Also, based on our results, we suggest an extension of the enactive theory of non-interactive social experience.

## Introduction

Empathy is central to intersubjective life. It is a critical component of our capacity to understand other people’s minds and to predict and explain their behavior (Stueber, 2019). Despite its importance in social life, the concept of empathy is still debated due to extensive associations with social phenomena (Cuff et al., 2016; Hall and Schwartz, 2019). For instance, some authors consider empathy essential for responding ethically to others (Caplan and Goldie, 2011), as well as for developing morality, prosocial action, and motivation (Hoffman, 2001; Decety and Cowell, 2014). On the other hand, other authors state that empathy is related to cruelty and immoral behavior (Decety and Cowell, 2018). Furthermore, empathy has even been considered a prerequisite for the scientific study of consciousness (Thompson, 2001) and the privileged method in social sciences (Dilthey, 1961).

A relevant aspect of studying intersubjective life is empathy for pain. Studies have consistently shown that exposure to pain images induces empathy and activates neural circuits similar to those triggered by first-hand experience of pain, particularly the brain regions linked to affective-motivational processing of pain. This evidence emphasizes the implicit and automatic neural representations shared between oneself and others to experience empathy for pain. This experience is generally aversive, unpleasant, and even painful for the observers themselves (Lamm et al., 2007, 2011; Bernhardt and Singer, 2012).

Although empathy is a core component of social life, there is no clear theoretical consensus on its definition. As Neumann et al. (2015: 257) point out, “an examination of the definitions of empathy in the last 20 years reveals that there is no single definition that is systematically quoted; in fact, the multitude of definitions is often quoted as a distinctive feature of the field.” The concept of empathy is also essential in various disciplines, such as social neuroscience, psychology, psychiatry, philosophy, and aesthetics.

In the field of social neuroscience, the most popular approaches to empathy are the theory-theory (TT) and the simulation theory (ST). Although both involve mind-reading, TT uses theoretical inferences while ST employs a first-person simulation routine (Keysers and Gazzola, 2006).

The theory theorists (Gopnik and Wellman, 1992; Baron-Cohen, 1995; Carruthers, 2009) consider that humans are capable of reading minds because they possess a common-sense “theory of mind” (ToM) with which they explain human behavior. As one of the most general terms in empathy research, ToM refers to the capacity to attribute mental states to oneself and others, and make predictions about others’ future behaviors by inferring their mental states (Premack and Woodruff, 1978). ToM maintains that the knowledge we acquire about our and others’ minds is not a formal scientific theory but an informal, everyday, or fundamental theory. For example, experience plays an essential formative role in developing the ToM in children (Flavell, 2004).

By contrast, simulation theorists (Gordon, 1986; Goldman, 2006; Gallese, 2009) deny that our understanding of others is theoretical in nature. Instead, they argue that we use our minds as a model for understanding others’ minds. Consequently, mind-reading depends on the ability to mentally simulate another person’s mind (Gallese and Goldman, 1998; Goldman, 2006). This perspective suggests that people perform internal simulations of the observed sensations, emotions, and actions of others (Oberman et al., 2007). For example, Uddin et al. (2007) suggest that we have specific brain networks for processing external (more bodily) actions and attitudes of ourselves and others through simulation, while other structures allow us to infer our and others’ mental states through ToM.

Neuroscientific findings on human mirror neurons have been interpreted as empirical evidence supporting ST. Mirror neurons have been considered acentral factor that enables the development of intersubjective relationships and the root of social cognition (Oberman et al., 2007; Stueber, 2019). Although the TT and the ST start from different theoretical assumptions, both are based on a representationalist perspective that presupposes one’s mind as internal, hidden, and fundamentally opaque to others. Consequently, others’ minds are not accessible from experience but through indirect processes such as theoretical inference or simulation (Zahavi, 2010; Colombetti, 2014).

As an alternative to these approaches, the phenomenological perspective offers a different explanation of the nature of empathy (Stein, 1917; Husserl, 1973; Scheler, 1973). For Husserl, “*Einfühlung*” [literally “feeling into,” translated by Titchener (1909) as “empathy”] is a particular form of intentionality in which one’s consciousness is directed toward another’s experience. He wrote that “in empathy, the empathizing I experiences the inner life or, to be more precise, the consciousness of the other I” (Husserl, 2006: 82). Husserl’s approach to empathy was further developed by his student Edith Stein in her 1916 doctoral thesis, *On the Problem of Empathy Stein (1964).* She affirms that observing the other’s subjectivity does not require explicit reasoning (“*Einsicht*”) but simply that we perceive and feel (“*Einfühlung*”). Consequently, Stein emphasizes the sensual experience of the empathic answer, which she terms “sensual empathy” (“*Empfindungseinfühlung*”). Stein’s proposal is the basis of the approach to empathy developed by Giovanna Colombetti (2014), who stresses the affective and bodily dimensions of empathy. As defined by Colombetti, basic empathy corresponds to our bodies’ affective response to others’ bodily presence. It is the most elemental experience of the other and takes place as soon as she enters our perceptive field. However, the other’s body does not appear simply as a physical object (“*Körper*”) but as a living body endowed with subjectivity (“*Leib*”), following the classic Husserlian distinction. In this study, we follow Colombetti’s phenomenologically inspired characterization of empathy and define it as affective and bodily “*experiential* access to the other’s subjectivity” (Colombetti, 2014: 174).

The enactive approach (Varela, 1984, 1991; Varela et al., 1991; Thompson and Varela, 2001; Thompson, 2007) is the explanatory framework in cognitive science that underlies Colombetti’s proposal and the present study. One critical contribution of this framework is placing conscious experience central to the scientific study of the mind. In *The Embodied Mind*, Varela (1991: 15) state that “the new sciences of mind need to enlarge their horizon to encompass both lived human experience and the possibilities of transformation inherent in human experience.” Moreover, following the phenomenological tradition, especially Merleau-Ponty’s development, they endorse the phenomenological view that considers the bodies of conscious creatures, especially human bodies, “both as physical structures and as lived, experiential structures—in short, as both ‘outer’ and ‘inner,’ biological and phenomenological” (Varela et al., 1991: 15).

Despite the importance of studying the subjective experience, most empathy studies have focused only on physiological mechanisms and used only self-reported assessments to investigate subjectivity (e.g., Timmers et al., 2018). Such subjective reports have been crucial to validating empathy for pain paradigms and relating physiological responses to changes in subjective responses (e.g., Klimecki et al., 2014). However, self-report methods have several limitations. For instance, self-report questionnaires quantify the participant’s empathic perception but do not describe how the subjective experience unfolds or how the interaction with another is experienced, thus overlooking a subtle subjective world (Olivares et al., 2015; Petitmengin, 2017). In addition, when these studies assess participants’ emotional state (e.g., fear and anger) or their empathic perception (e.g., valence and arousal), it is assumed that they must be and have been aware of the questions to which they are subjected, having the risk of inducing or colluding a subjective state (Hurlburt and Heavey, 2015). Another limitation is that self-report assessments reveal previous theoretical assumptions about the nature of empathic experience, and thus prevent knowing the participant’s subjective in their words and experiential domains (Colombetti, 2014).

In contrast to self-report methods, phenomenological methods aim to understand the structures of human experience, including highly embodied dimensions and multi-layered, intricate dynamics of lived experiences (Bitbol and Petitmengin, 2017). Since its incorporation into the field of cognitive science, phenomenology has exhibited accuracy and high utility in understanding phenomena such as meditative states (Silva-Mack et al., 2018; Nave et al., 2021), contemplative training (Przyrembel and Singer, 2018), epilepsy (Le Van Quyen and Petitmengin, 2002), fibromyalgia (Valenzuela-Moguillansky, 2013), and chronic pain (Smrdu, 2022). In addition, analysis of subjective experience captures very subtle descriptions of embodied experiential microdynamics, such as approach and avoidance behaviors (Baquedano and Fabar, 2017), exploration of awareness during sleep (Alcaraz-Sánchez et al., 2022), adjustments of attention (Lachaux et al., 2000), variation of emotional state (Depraz et al., 2017), and movement intention (Jo et al., 2014).

To the best of our knowledge, no prior research has incorporated subtle descriptions of empathic experience. However, a series of promising studies have classified participants according to the experience of consciously feeling vicarious pain in a classic empathy for pain paradigm. Notably, these studies show differences in functional connectivity among the subjective clusters (Grice-Jackson et al., 2017a,b). Although these studies analyzed subjective experience through self-report questionnaires and considered few bodily and affective subjective dimensions, they reveal the significant contribution of incorporating the study of subjective experience into paradigms of empathy for pain.

This study aims to deepen the knowledge of empathy for pain by implementing an experimental phenomenological method, which entails “discovering the structure of the experience as it appears in consciousness through a research design that incorporates the peculiarities of experimental psychology and phenomenological psychology” (Martínez-Pernía, 2022: 149). To achieve our goal, we examined the phenomenological experience with an emphasis on embodied, multi-layered dimensions (bodily sensations, emotions, and motivations) and temporal aspects of empathic experience. Our research design exposed participants to a video of people having physical accidents while practicing extreme sports. We applied a second-person method (phenomenological interview) to rigorously collect phenomenological data (Petitmengin, 2006; Olivares et al., 2015; Petitmengin et al., 2019), which we analyzed by drawing on Giorgi’s phenomenological analysis (Giorgi et al., 2017).

The study’s relevance regarding social emotions, and more specifically empathic experience of pain, lies in two central elements. First, social cognition research has traditionally been conducted through self-report, behavioral, and neuroimaging measures (Neumann et al., 2015), but first-person methods based on the phenomenological experience have been neglected^1^ (Gallagher, 2011; Fuchs, 2013). We will also discuss how phenomenological data may contribute to comprehending empathy for pain in social neuroscience. Second, since the enactive approach emerged, just a few of the plethora of publications in basic science have implemented enactive concepts with qualitative research (Fernandez, 2020). This article discusses our phenomenological results as a resource to explore the unfolding of experience from the perspective of the enactive approach (Stilwell and Harman, 2021; Martínez-Pernía, 2022).

## Materials and methods

### Participants

Between September 2017 and January 2018, 28 adults participated in the study. Inclusion criteria required individuals with no clinical history of cognitive, neurological, or psychiatric disorder and normal or corrected-to-normal visual acuity. Criteria were corroborated in a brief interview. To characterize the sample, participants were asked to complete the Montreal Cognitive Assessment (MoCA; Nasreddine et al., 2005), the Beck Depression Inventory-II (BDI-II; Beck et al., 1996), and the State-Trait Anxiety Inventory (STAI; Spielberger et al., 1970).

Participants were recruited from workers and university students at Hospital del Salvador (Santiago, Chile). All participants gave written informed consent. The study procedure was conformed with the Declaration of Helsinki principles and was approved by the “Scientific Ethics Committee of the Servicio de Salud Metropolitano Oriente” and the “Research in Humans being Ethics Committee of the Medicine Faculty, Universidad de Chile.”

### Construction and validation of the emotional stimuli

To construct and validate the stimuli, we followed the methodological considerations widely used in affective neuroscience (e.g., Stemmler, 2003). Empathy for pain stimuli were produced using audiovisual material found online under Creative Commons licensing. In total, 12 scenes that included men and women, with an average duration of 7–11 s, were used to validate the emotional condition. Each scene depicted an intense physical accident resulting from wrong movements or miscalculations while practicing extreme sports (e.g., parkour, skateboarding, snowboarding, or climbing). All 12 scenes had a similar event sequence. Each began with a sportsperson skillfully practicing a sports activity. The sportsperson then loses balance and impacts heavily against the ground. Finally, the sportsperson is seen lying on the ground. No scenes depicted dismemberment, disfigurement, or death.

Once all the scenes were prepared, they were validated with 65 university students (38 women; mean age = 19.34±1.56) following the indications of the Self-Assessment Manikin (Bradley and Lang, 1994). This scale assesses the person’s emotional reaction to the stimulus in terms of valence (“unpleasant” to “pleasant”), arousal (“low” to “high”), and dominance (“without control” to “with control”) on a 9-point rating scale (1–9). Empirical works have repeatedly confirmed that these emotional dimensions effectively measure a person’s affective reaction (Bradley and Lang, 1994). Higher scores indicated pleasant valence, more arousal, and having control of the situation; lower scores indicate unpleasant valence, less arousal, and losing control of the situation. Because this article aimed to study empathy for others’ physical pain, we selected as the final scenes those scored in the trial as unpleasant, provoking high arousal, and triggering the perception of lost control. The selected scenes had the following mean scores: 3.77 (±1.94) for valence; 6.40 (±1.78) for arousal, and 5.31 (±2.68) for control. Seven scenes (six men and one woman) were combined to produce the final video (60-s duration) that all participants in the main study would watch (the video was uploaded^2^).

### Procedure

Participants completed the questionnaires and all the experimental protocols in the Clínica de la Memoría y Neuropsiquiatría (CMYN) del Hospital del Salvador. A psychologist supervised the informed consent process and verified that each participant met the inclusion criteria through interviews and the previously described scales. After this step, we implemented an experimental protocol previously used in affective neuroscience research (e.g., Hagenaars et al., 2012, 2014; Gea et al., 2014). Each participant was requested to stand on a marked spot exactly 1 m from a 40-inch screen TV, installed at eye level.^3^ They had to motionlessly maintain a comfortable bipedal stance, with their arms relaxed alongside the body. The video was then played on the screen. Immediately after it finished, a researcher conducted a phenomenological interview with each participant, explaining the aim of this kind of phenomenological inquiry. Details of this interview are presented in the next section.

### The phenomenological interview

The same researcher conducted all 28 phenomenological interviews in Spanish (DM-P). They were audio-recorded and later transcribed verbatim. At the beginning of each interview, the participant was asked to describe the scenes that induced unpleasant feelings and then choose the scene (participants chose all different scenes) associated with the highest overall intensity of their experience (no participant had difficulty identifying this). This process enabled the whole interview to focus on the singular experience of the selected scene.

The researcher who conducted the interviews adopted the phenomenological attitude, also named “epochç” (Moustakas, 1994; Merriam, 2009). It allows studying the appearance of the phenomenon as such, through suspending the natural attitude with which we usually know and the step toward a phenomenological attitude. Phenomenological reduction leads to the source of meaning and existence of the experienced world (Moustakas, 1994). The interviews were also partially guided by the criteria for micro-phenomenological interviews (Petitmengin, 2006). In order to help the participant to assume the phenomenological attitude, the interviewer maintained the principle of evocation during the interview (Petitmengin et al., 2019). Implementing the principle of evocation is essential to obtain the participants’ pre-reflective descriptions and to make their past experiences more vivid (Petitmengin et al., 2019). This is important because “usually interviewees glide into general descriptions of condensed situations that make it difficult to produce precise descriptions. Therefore, it is important to continually bring the interviewee back to the chosen particular situation” (Valenzuela-Moguillansky, 2013: 340–341). Below is an example of how this part of the micro-phenomenological interview method was implemented:

*“…Well done [name of participant]. Now, I will ask you to close your eyes, and visualize, feel as if you are re-living the experience of watching the video. So, close your eyes please, and see yourself again in this situation in which you are standing, watching the television screen, and the accidents occur…”*

Another relevant aspect of the phenomenological interview was to collect data conveying “what” the participant experienced and “how” she experienced it, for example, by asking, “What do you feel?”; “How do you perceive it?”; and “How do you know it?” The time course of the experience was also taken into account, for example, by asking, “How do you feel at the beginning of the video?”; “And just after that, how do you feel?”. Another characteristic of the interview procedure was recapitulating participants’ responses to facilitate their recalling.

### The descriptive phenomenological psychological method

To phenomenologically analyze the data, we used the descriptive phenomenological psychological method, hereafter Giorgi’s method (Giorgi, 1975; Giorgi et al., 2017). This method centers analysis on the meaning of the experience and aims to describe its structure by identifying central themes (Giorgi et al., 2017). In this sense, the experience’s psychological structure refers to how the subject makes sense of her own lived experience in the world. This method considers the experience as a psychological consciousness through a non-transcendental phenomenological psychological method (Giorgi, 2021).

Each of the three researchers involved in data analysis (DM-P, AT, and KB) began by reading an interview in full and then summarizing its general meaning. We then carefully re-read the interview, highlighting in the transcribed document every statement expressing or referring to the direct experience (meaning units). After completing this, we re-read each meaning unit to identify the sub-themes and main themes in which the lived experience occurred. For this, the researchers must transform the participant’s expressions into categories that highlight their psychological meanings. It requires maintaining the original meaning of singular verbatims while simultaneously allowing a generalization of them to similar experiences of other participants (Giorgi et al., 2017). The last step of Giorgi’s method is to grasp and describe the whole structure of the experience. Because the main themes inform us about specific parts of the experience, we tied the main themes together to get a whole structure. To achieve this structure moving from particular aspects to participants’ essential understanding, we looked at these particular elements and systematically varied them to determine their psychological essence. It is important to clarify that the main themes characterizing the specific aspects of the experience emerge in the penultimate step of the analysis process, while the whole psychological structure of the experience emerges in the last step. The qualitative results will be presented in this order in their corresponding section.

After completing their individual analyses of each interview, the three researchers met to triangulate the data by jointly reviewing each analysis. We compared and discussed the meaning units, themes, and essential structure of the interview during the triangulation process. Any disagreement had to be resolved by reaching a consensus among the researchers. Our analysis also used iteration throughout the procedure. Where a new main theme or sub-theme appeared or was modified, we had to review all previous analyses to keep consistency between the new and previous categories. This review procedure and consistency were also implemented in the structural experiential analysis.

The individual and triangulation analyses were supported by ATLAS.ti (2022) 9 qualitative data analysis software and implemented for each of the 28 interviews.

### Quality assurance

We deployed several measures to ensure the quality of data collection and analysis. First, the researcher who conducted the interviews, who is certified in a micro-phenomenological interview, adopted a phenomenological attitude by setting aside or bracketing beliefs, prejudgments, and thoughts. Some examples of these mind processes that had to be set aside were: the researcher’s inferences that his own past experiences are the same as that the participant is living, or presuppositions that specific experiences of the participant are understandable from a pre-established theoretical model. For the purpose to adopt the phenomenological attitude, the interviewer disclosed his own evaluations and experiences before data collection, aiming to be open to observing emerging phenomena without preconceptions (Hamilton et al., 2018). Moreover, before and during each interview, the researcher deliberately examined his own beliefs and their temporary suspension, being open to observing emerging phenomena without preconceptions (Hamilton et al., 2018). This phenomenological attitude was also maintained rigorously by all three researchers throughout the data analysis process. In addition to adopting the phenomenological attitude, the researchers “reduce” or restrict their frames of reference to the psychological meaning (psychological reduction). This means that they have to focus on a dimension of the experience that “is neither abstractly conceptual, nor objectively physical; it is concretely and personally lived, by a particular person, always socially engaged, in a particular situation in everyday social life, in space, time and history” (Englander and Morley, 2021).

As a second quality-assurance measure, the three researchers are all experienced in phenomenological studies and each independently analyzed the 28 interviews using ATLAS.ti (2022) 9 qualitative software.

As a third measure, we implemented a four-step quality procedure to ensure the reliability of triangulation analysis for sub-themes in each temporal phase and the experiential structure. In the first step, the three researchers jointly analyzed the first ten interviews in a systematic and rigorous triangulation process, in which we corroborated the different phenomenological categories generated for every utterance by each participant, together with the underlying experiential and linguistic criteria used by each researcher separately to generate the phenomenological categories. After reaching a consensus in these analyses, the researchers shared a common view about the analyzed interviews and phenomenological categories. In the second step, to triangulate the last 18 interviews after independent analyses, each researcher downloaded the ATLAS.ti software the quantitative data in XLS file format with the phenomenological coding of each interview. The codings of the three researchers were then displayed in the R statistical programming environment to reveal which phenomenological categories were agreed upon or subject to disagreement. In the third step, the researchers identified any category for which there was no consensus that undertook the same systematic, rigorous triangulation process described in the first step. Finally, the fourth step entailed analyzing inter-rater agreement using Fleiss’ Kappa on each sub-theme and each individual phase. This calculation was performed only in independent categories (e.g., multifocal). This coefficient calculates the level of agreement of inter-raters on categorical data and is considered more reliable than a simple calculation of the agreement ratio (Fleiss et al., 2013). This calculation allows for analyzing the inter-rater agreement and provides feedback to detect errors, omissions, and disagreements among the researchers. If a kappa below 0.8 was observed, we went back to the previous steps. The average kappa was 0.97 (0.85–1.00) (for more detail, see text footnote 2). Finally, the experiential description for each participant was performed with a common data set containing a complete agreement and common quotes.

### Quantitative analysis

Once the phenomenological analysis was completed, quantitative analyses were conducted in the *R* statistical programming environment. First, we calculated the number and percentage of participants for each main theme and sub-theme. We also studied the phenomenology of empathy for pain through a system-thinking perspective (Meadows, 2008), designing a relationship map based on network analysis (Newman, 2010; Barabási, 2012). This analysis allows us to focus on independently studying experiential categories and how phenomenological categories interact and create relational patterns with other experiential categories, which are represented in a network analytical diagram. For this qualitative approach, we elaborate a network diagram using the software IBM SPSS v28.0. This diagram uses statistical frequency analysis to calculate the size of each node based on the frequency of subjects belonging to that experiential categorization and its associative co-occurrence with other categories. In this case, the co-occurrence considers the frequency in which the subjects appear within each phenomenological category defined in the data set, grouping them as a network. Nodes represent phenomenological categories, while links represent the strength of influence between them. Larger nodes and thicker lines, respectively, represent stronger influence and connections. Conversely, smaller nodes and thinner lines, respectively, represent weaker influence and connections.

In summary, implementing these methodological procedures (qualitative and quantitative analyses) will allow us to show our results from two research approaches. On the one hand, we will formulate a detailed phenomenological description of the empathic experience of pain. On the other hand, we will show how the descriptive results are understood from an analytic view.

## Results

### Participants

Twenty women and eight men participated in the study (mean age = 29.6 ± 6.6 years; mean years of education = 16.9 ± 2.4). The participants report a MoCA total: mean = 28.5 ± 1.5; STAI total: mean = 48.8 ± 12.5; BDI-II total: mean = 5.0 ± 5.7 (for more detail, see text footnote 2). Two participants showed scores for depression (29) and cognitive alteration (24) far away from their normative reference group. Their phenomenological results were similar to the rest of the participants.

### Phenomenological results

**FIGURE 1:**
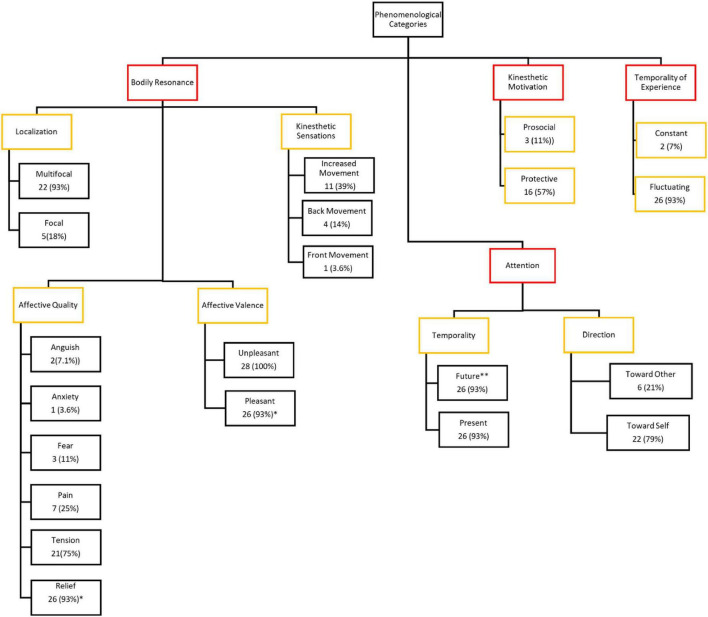
Representative scheme of the experiential categories found in empathy for pain. Categories in red show the main themes, categories in yellow show the sub-themes, and categories in black show sub-categories. *N* (%) represents the number and percentage of participants who described the experience. *Experience specifically identified in the recovery phase. ^**^Experience specifically identified in the anticipatory phase.

The description of the empathic experience of pain was extracted from 28 interviews. Each full interview lasted 15 min on average, and the total interviewing time was 420 min. In the first abstraction level of the coding procedure, we identified 42 meaningful codes (e.g., heart palpitations). These codes were then transformed into 21 emergent experiential sub-categories (e.g., multifocal sensations). Next, experiential sub-categories were grouped into sub-themes with a similar thematic affinity (e.g., localization of bodily sensations). Finally, four main themes were identified at the maximum abstraction level: bodily resonance, kinesthetic motivation, attentional focus, and temporality of experience (Figure 1). These four main themes are present in all 28 experiences examined; however, they manifest differently in the two types of experiential structures: self-centered empathy for pain and other-centered empathy for pain (this description will be shown in the experiential structure section).

### Phenomenological description

This section describes the four main themes identified in participants’ experience and the categories that compose them (for a more complete and holistic understanding, refer to the Codebook at see text footnote 2).

#### Bodily resonance

While watching the video, participants’ bodily experiences resonated in coordination with the sportsperson’s bodily actions. Bodily resonance means that the participant is bodily affected by the sportsperson’s behaviors. Thus, participants’ bodily resonance was intertwined with the sportsperson’s movements, producing a wide range of bodily, affective, and kinesthetic sensations according to the events and actions of the sportsperson.

Concerning bodily sensations, participants felt diverse sensations in muscles and viscera, either focused on a specific bodily region (focal, 18%) or several regions simultaneously (multifocal, 93%). The specific parts where participants felt sensations were the abdomen (36%), chest (50%), heart (32%), face (11%), lower extremity (21%), and upper extremity (39%). These bodily sensations were related to the upcoming fall event or the sportsperson’s fall.

“Yes, yes… and when the person hits the ground I feel even more pressure in my stomach.” (P19)

“When the person has already fallen, the tension in the gut does not persist, it starts to decrease until it disappears… or I don’t know if it completely disappears, but it definitely decreases.” (P13)

Together with the bodily sensations, several negative emotions emerged during participants’ experiences intertwined with the event that the sportsperson was living. All participants described discomfort that made them feel unpleasant emotions while watching the video. They verbalized these emotions as “*tension*” (79%), “*pain*” (29%), “*fear*” (18%), “*anguish*” (18%), and “*anxiety*” (7.1%). Toward the end of the scene, when the sportsperson had already suffered the accident, most participants (57%) also felt an emotion of relief, which they described as recovering to their normal state.

“Yes, my tension keeps increasing a lot according to how she [the sportsperson] advances, because I could anticipate what was going to happen, I said, ‘Something bad is going to happen,’ so, as she progresses, I feel more sensitivity, more pressure in my chest, I breathe a lot as the video progresses, it increases, I breathe a lot…” (P20)

*“Once the person falls, the tension dissolves. The person starts to fall, and the tension starts to decrease, I know it is impossible that he dies, but it is interesting because the tension dissolves.”* (P19)

The third dimension of bodily resonance is kinesthetic sensations, experienced by many participants (46%) as feeling “unbalanced” (46%) as their bodies autonomously reacted to the unfolding scenes, which induced the feeling of “losing” bodily control.

*“I got a bit unbalanced when I saw a fall.”* (P10)

#### Kinesthetic motivation

Participants reported that, while watching the video, they experienced the “*feeling*” in their bodies, or sections of their bodies, of “*wanting*” to do something concerning what was happening in the video. Most (54%) described the motivation to avoid or escape from watching the video.

*“I almost turned my head… No. I did nothing, I did nothing, but I had the intention; it was the first thing that came to me like this.”* (P11)

*“And the feeling of rejection, so I kind of leaned my body back, and I also tried to frown a lot, as with that feeling of… I don’t know, rejecting it, as my whole body and all my movements that do kind of line up, as if trying to get away from, from that video.”* (P28)

*“A feeling of wanting to get away, as if my body was going backward.”* (P16)

A few participants (11%) reported feeling the need to help the sportspersons avoid suffering any injury while practicing extreme sports.

*“Hold them down, so they don’t do something stupid like, they are going to kill themselves or get injured. Or wanting to do something to avoid the situation.”* (P22)

*“I felt that, if I did move in some way, I was going to prevent them from falling, as if my corporeality could be… as if I was getting involved with them.”* (P5)

#### Attentional focus

In addition to this whole bodily, affective, and motivational experience, participants’ attentional focus comprised directional and temporal dimensions while watching the video.

Concerning the direction of attentional focus, most participants (79%) centered their attention more on their own personal discomfort and sensations of rejection while watching the sportspersons.

*“Fear, I want to move, to protect myself.”* (P11)

*“But, it’s like a rejection more than anything. It’s something I don’t necessarily want to watch.”* (P9)

The remaining participants (21%) centered their attentional focus primarily on the pain and suffering experienced by the sportspersons.

*“What I feel the most is tension … Thinking no, I hope he doesn’t fall, but knowing that he is going to fall.”* (P3)

*“Like… nervousness. I couldn’t do anything to prevent the falling.”* (P24)

Regarding the temporal dimension of attentional focus, some participants described the events unfolding in the video with reference to the future while others referred to the present. Participants with future-directed attentional focus described expectations of the immediate and extended future, preoccupation about what might happen to the other, negative judgments about the other’s decisions, and beliefs that something catastrophic might happen as a consequence of the accident.

*“Like, you get into the video, as it is there, anticipating something that I am seeing is going to happen.”* (P4)

*“I knew she was going to fall, but I did not know how she was going to fall, what was going to happen.”* (P11)

Conversely, participants with present-directed attentional focus described the events the sportspersons were living at that precise moment. Some descriptions related to the sportspersons’ movements to maintain balance or to the impact of their bodies on the floor.

*“I hope it wasn’t so serious. I thought, ‘Oh, what pain!”’* (P3)

*“It annoys me a little because she does something completely absurd, which is to stretch out a leg, which has no purpose other than to fall, and, well, she falls.”* (P12)

#### Temporality of experience

During the video, most participants (93%) perceived a temporal fluctuation in their experience, identified in three different temporal moments: anticipatory, climax, and recovery. By contrast, the remaining participants felt no temporal change in their experience as the video unfolded (7%).

The first temporal moment of most participants’ experience was heavily characterized by a sense of anticipation of the looming accident, with a gradual increase in the intensity of their negative emotions, bodily sensations, and thoughts as each scene progressed: the longer participants watched the sportsperson, the more intense their experience became.

*“My tension is increasing a lot according to how the video progresses because I could anticipate what was about to happen.”* (P18)

The second temporal moment of the experience began just a few instants before the accident happened and included the time of the accident occurring. In terms of their bodily resonance and *kinesthetic motivation*, participants’ experiential intensity climaxed during this moment.

*“[referring to the rise in unpleasant bodily sensations] When the impact with the ground occurs, that is the moment when I feel the most intensity.”* (P26)

In the final temporal moment that followed the accident, with the observed sportsperson already lying on the ground, participants’ experiential intensity tended to diminish significantly. Specifically, they felt a physical relaxation of their body accompanied by the easing of negative emotions, bringing relief, tranquility, and less concern.

“*This person is putting himself more and more in a risky situation while climbing, and once he has already fallen, I feel relieved…” (P12*)

### Experiential structures

**FIGURE 2:**
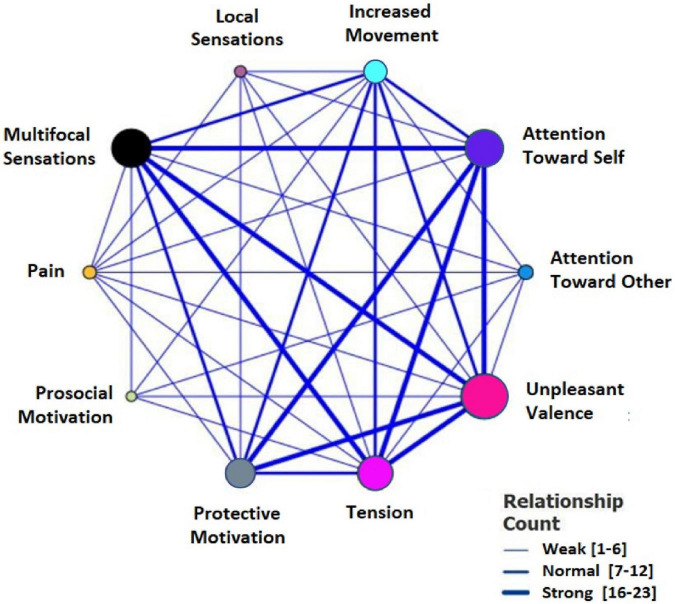
Relationship map of the phenomenological categories.

The four main themes elaborated above are the main elements of the experiential structures arising from our data analysis. Although these phenomenological categories are described as autonomous phenomenological dimensions, they are tightly intertwined in participants’ experiences. As shown in the relationship map (Figure 2), the different sub-themes and sub-categories closely interact, showing that the empathic experience is a holistic and complex process of interaction between corporeality, affectivity, kinesthetic motivation, and the direction of attention.

However, these phenomenological categories did not appear in an equal manner in all experiences. The way they presented themselves in the experiences of different participants was very distinct; in this sense, two experiential structures were identified: self-centered empathy (*N* = 22) and other-centered empathy (*N* = 6) (for details of the quantitative analysis between empathic structures, see text footnote 2). At the core of both structures, we found the direction of attentional focus and kinesthetic motivation. Attentional focus expresses where participants’ concern and affective quality are directed (at themselves or the sportsperson), while kinesthetic motivation is participants’ pre-reflective intention to self-protect or help the sportspersons.

#### Self-centered empathy

**FIGURE 3:**
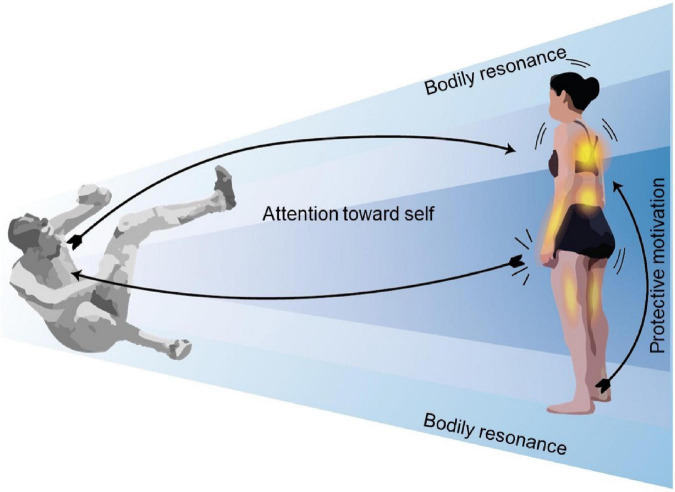
Experiential structure of self-centered empathy for pain. The empathizer’s intentional object is the empathized experience; they see the sportsperson as sentient and having a painful experience. This empathic structure is characterized by bodily resonance, attentional focus centered on the participant’s own experience of seeing the other suffer, and self-protective kinesthetic motivation.

Participants who showed this experiential structure were preoccupied with the other and had intense feelings of discomfort but mainly focused on themselves. Although they referenced the pain suffered by the sportspersons, they focused on their own emotions of discomfort and how these emotions made them upset and uncomfortable. This was accompanied by a general sensation of rejection toward the video, which manifested affectively motivationally as a “feeling” of not wanting to look, or “wanting” to turn away. Their preoccupation with the other and feelings of discomfort also involved the activation of different bodily sensations while watching the video, such as muscular sensations (e.g., tension in the arms, legs, and trunk) and visceral sensations (e.g., palpitations, breathing, and oppression in the chest). Overall, participants’ attentional focus was directed toward the other person having a painful fall but their kinesthetic motivation and affective levels were primarily centered on themselves (Figure 3). Regarding the temporal dimension of the experience, participants perceived mild bodily sensations and feelings at the start of the video, but these tended to intensify as each scene unfolded, reaching a peak when the fall occurred. After that moment, participants felt their bodily resonances began to decline.

#### Other-centered empathy

**FIGURE 4:**
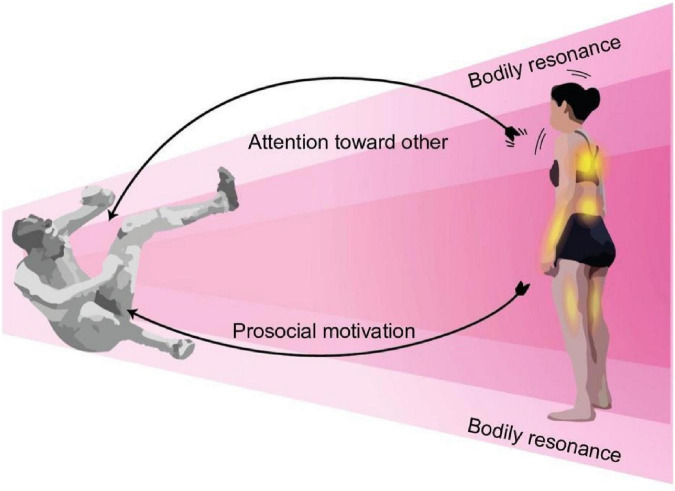
Experiential structure of other-centered empathy for pain. The empathizer’s intentional object is the empathized experience; they see the sportsperson as sentient and having a painful experience. This empathic structure is characterized by bodily resonance, attentional focus centered on the sportsperson, and prosocial kinesthetic motivation to help him.

Participants who exhibited this experiential structure were greatly preoccupied with what was happening to the sportspersons in the video. They needed to find ways to help the other, which manifested in physical and verbal potential actions directed toward them: to “*grab*” the person so they would not fall or shout to alert them of what lay ahead. Their preoccupation also involved different muscular sensations (e.g., tension in the arms, legs, and trunk) and visceral sensations (e.g., palpitations, breathing, and oppression in the chest) while watching the sportspersons. Overall, these participants’ attentional focus was more centered on the sportspersons and their kinesthetic motivation was to avoid their suffering (Figure 4). In addition, the temporal development of their experience was similar to that described for self-centered empathy.

### Quantitative analysis of the experiential structures

**FIGURE 5:**
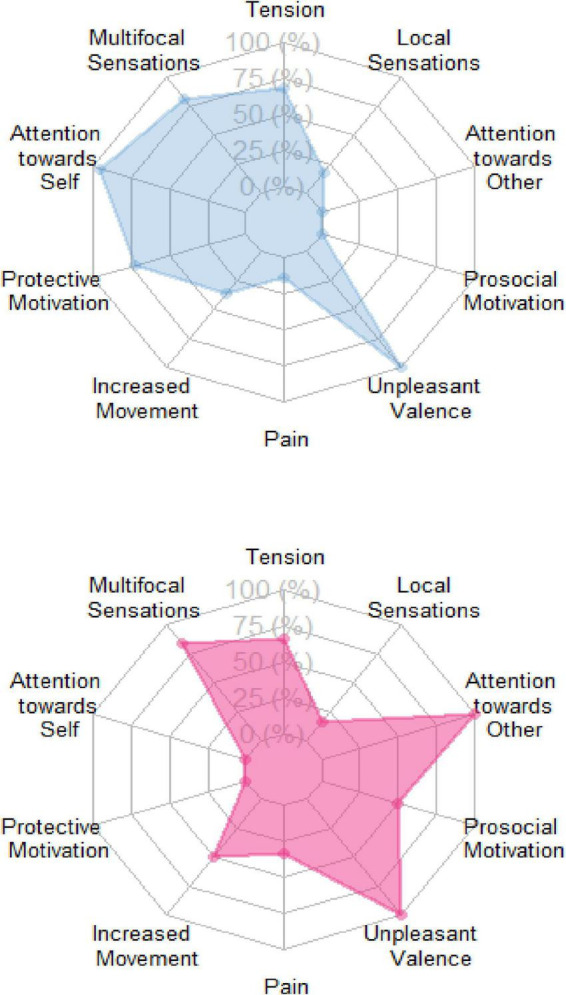
Quantitative analysis of the experiential structures of empathy for pain. The figures show the frequency (%) of participants in the self-centered empathy for pain (blue) and other-centered empathy for pain (pink) groups experiencing each phenomenological category.

The two empathic structures share similar phenomenological categories in the bodily resonance, affective, and temporal dimensions (for more details, see text footnote 2). Nonetheless, self-centered and other-centered empathy differ in the phenomenological categories of attentional focus direction and kinesthetic motivation. Thus, participants with the other-centered empathic experience (*N* = 6) directed their attention toward others (100%) and manifested a prosocial kinesthetic motivation to help the sportspersons (50%). Conversely, participants with the self-centered empathic experience (*N* = 22) directed attention to themselves (100%) and manifested a kinesthetic motivation to avoid or escape from the sportspersons (68%). The quantitative differences in the experience of phenomenological categories between other-centered and self-centered empathy are highlighted in Figure 5.

## Discussion

Through an experimental phenomenological method, this study explored the phenomenological experience with an emphasis on embodied, multi-layered dimensions (bodily sensations, emotions, and motivations) and temporal aspects of empathic experience. Similar to other theoretical studies (Fuchs, 2013; Fuchs and Koch, 2014), our results show that experiential access to the other person’s painful experience involves a multiplicity of bodily sensations and negative emotions that fluctuate through time, accompanied by various kinesthetic sensations and motivations. In contrast to other empirical studies deploying first-person methods (Grice-Jackson et al., 2017a,b), every participant reported perceiving bodily sensations during the empathic experience of pain. More specifically, we found that bodily resonance was central to participants’ experience and was described in great detail, comprising muscular sensations (e.g., tension in the arms, legs, and whole body) and visceral sensations (e.g., palpitations, breathing, and oppression in the chest), negative emotions (e.g., tension, pain, fear, anguish, and anxiety), and involuntary backward/forward swaying of the body. In addition, participants’ experiences while watching the video showed directional and temporal dimensions and involved the feeling of their bodies wanting to do something concerning events unfolding on screen. Finally, participants witnessed temporal fluctuations in their experiences, bringing intensity changes in their bodily sensations, emotions, and motivations.

Our analysis identified two experiential structures: self-centered empathy for pain and other-centered empathy for pain. These two structures differ mainly in the direction of attentional focus and kinesthetic motivation. In the case of self-centered empathy, participants focused attention on their own unpleasant experience of watching the accidents unfold, and their behavioral motivation was to avoid or reject. By contrast, in the case of other-centered empathy, participants focused attention on the sportspersons’ harm and suffering, which elicited the motivation to help through physical or verbal action. Similarly, it is relevant to mention that although the experiential structures are mainly characterized by two independent phenomenological categories (direction of attentional focus and kinesthetic motivation), other categories were shared in both structures (e.g., bodily sensations, affective quality, and increased movement). These results suggest that the empathic experience of pain embraces an intertwined emotional continuum, ranging from self-centered empathy to other-centered empathy. This means that participants are not experiencing two fully dichotomous or independent structures. Instead, they are living an empathic experience constituted by shared embodied categories. Although our findings did not show that self-centered and other-centered empathy shared the main categories that characterized these structures (direction of attentional focus and kinesthetic motivation), it is relevant to mention that this study was implemented with a restricted number of participants, reducing the possibility of findings new phenomenological categories or, even showing a more complex view of empathy (e.g., participants in the initial phase of the experience feel a self-protective motivation, but those in the climax phase feel the necessity of helping the sportspersons avoid falling to the ground).

The rest of the discussion is organized into two subsections. First, we will discuss how our phenomenological data contribute to comprehending empathy for pain concerning the neurobiological and behavioral data from social neuroscience. Because research in this latter field is central to our scientific understanding of social-emotional phenomena such as empathy discussing the implications of the current study to social neuroscience can have a significant positive impact in advancing and enriching the way in which empathy is typically approached, with the potential of gaining deeper, more accurate, and more useful scientific knowledge of this target phenomena. The second subsection will then discuss the implications of our results for the enactive approach to social-emotional experience. More specifically, we will address the phenomenological aspect of empathy for pain in the context of the three *dimensions of the embodiment* of human consciousness (Thompson and Varela, 2001; Varela and Thompson, 2003). The enactive approach is one of the main research programs in cognitive/affective science that has systematically given the body and lived to experience a central place in the study of cognition, perception, affect, intersubjectivity, and agency (Varela et al., 1991; Varela, 1996; Thompson, 2007; Fuchs and de Jaegher, 2009; Colombetti, 2014; Di Paolo et al., 2017). Thus, discussing the implications of our study for the enactive approach can contribute to advancing this important research program and with that, our scientific understanding of the key role that the body and subjective experience have in empathy and more generally, in social cognition, emotions, and intersubjectivity.

### Contribution of the phenomenological data to understanding empathy for pain in social neuroscience

The classic cognitivist approaches (TT and ST) and the phenomenological tradition show a problem of incommensurability; that is, they advocate incompatible ontologies and epistemologies (Small, 2011). For instance, the cognitive perspectives claim that private mental states inside the brain fundamentally constitute the mind while the body is an objective and public physical machine causally interacting with those mental states. Conversely, in the phenomenological tradition, the body is taken to be a sentient lived body and the mind is considered fully present in the meaningful arrays of facial gestures and body motions expressing the first-personal character of lived experiences (Thompson, 2007). Although the cognitive and phenomenological perspectives show contrary views at their conceptual roots, there is scope for dialog between empirical findings from studies applying these different approaches, which can improve comprehension of a given mental phenomenon (e.g., Morgan, 2007; Denscombe, 2008). This subsection will show how our phenomenological data contribute to comprehending empathy for pain in the field of social neuroscience.

There is now extensive evidence that the somatosensory and motor cortices are activated when seeing the pain in others (e.g., Hoenen et al., 2015; Fabi and Leuthold, 2017; Motoyama et al., 2017; Riečanskı et al., 2019). These findings have profoundly influenced comprehension of empathy, with some authors claiming that sensorimotor responses are the basic process of social cognition (Gallese, 2005; Hari et al., 2015) since social interaction occurs through exchanging sensations and movements with another. Concerning our phenomenological findings, participants felt a multiplicity of bodily and kinesthetic sensations. Muscular sensations were experienced throughout the body, thus occupying a central place in the experience (e.g., tension in the arms, back, legs, and hands). Participants also reported kinesthetic sensations like feeling “unbalanced” or “losing” bodily control. In addition, they felt motivated to move to either help the sportspersons or run away (kinesthetic motivation). An open question for future research is how these phenomenological reports are related to the brain processing of empathy. Specifically, studies should investigate whether these sensorimotor experiences are related to sensorimotor activity in areas of the brain considered as earlier (bottom-up sensory processing) (Riečanskı and Lamm, 2019), or to more complex empathic responses, such as the affective aspects and motivations of others (Prochazkova and Kret, 2017).

Another aspect to discuss concerning bodily sensations is interoception. Recent studies have shown an association between the perception of inner corporeal states and empathy (e.g., Fukushima et al., 2011; Terasawa et al., 2014). For instance, Ernst et al. (2013) found that activity in the bilateral anterior insula during an empathy task was enhanced when participants briefly attended to their heartbeats. In another study, Grynberg and Pollatos (2015) found that individuals who are able to more accurately perceive their own corporeal inner states (interoceptive sensitivity or interoceptive accuracy) felt higher pain intensity and more compassion in response to others suffering pain. These studies show that interoceptive awareness not only plays a crucial role in regulating homeostatic functions (e.g., thirst, hunger, and visceral urgency) and emotional awareness (Barrett et al., 2004; Wiens, 2005) but is also connected to sharing with other people’s emotions, such that people with better interoceptive awareness have greater empathic abilities, such as more compassion (Grynberg and Pollatos, 2015; Arnold et al., 2019). A possible neurobiological explanation for this relationship is that observation of another’s pain (Decety, 2010; Lamm and Singer, 2010) and interoceptive awareness (Craig, 2002) both depend on the activity of similar neuroanatomical structures (anterior insula and medial/anterior cingulate cortex). Concerning our phenomenological results, we identified an association between interoceptive awareness and the empathic experience of pain. Participants in both experiential structures reported abundant sensations in muscles (e.g., tension in the arms, legs, and back) and viscera (e.g., palpitations, changes in breathing rhythm, oppression in the chest, and pressure in the stomach). Future research could improve the understanding of interoceptive awareness concerning empathy. Traditionally, studies have implemented heartbeat-detection tasks or focused on the objective perception of heartbeats (e.g., Grynberg and Pollatos, 2015). However, knowledge is still seriously lacking on what it is like to experience the inner body states of empathy. Implementing experimental phenomenological methods could help improve this understanding.

Although empathy has been extensively studied through neuroimaging, it is also connected to physiological functions, such as postural control and autonomic response (Lelard et al., 2019; Jauniaux et al., 2020). Postural control studies report contradictory results concerning the effects of social situations on motor control. For instance, some studies show that aversive social stimuli (e.g., mutilation images) produce a decrease in postural sway, namely, a freezing response (e.g., Azevedo et al., 2005; Codispoti et al., 2008; Hagenaars et al., 2014), while others show that such stimuli provoke an increase in body sway (e.g., Gea et al., 2014; Brandão et al., 2016). Studies have also found that negative social emotions provoke withdrawal behavior motivated by self-defense (Lelard et al., 2019). Our phenomenological results show some evidence related to kinesthetic sensations and motivations. For instance, some participants reported feeling bodily imbalance, suggesting that their postural control responses brought an increase in body sway, similar to the findings of Brandão et al. (2016) and Gea et al. (2014). In addition, participants with self-centered empathy reported feeling motivated to avoid or escape from watching the video, suggesting they had a withdrawal behavioral response. Conversely, participants with other-centered empathy reported feeling motivated to help the sportspersons through cooperative behavioral responses, which is similar to Gea et al.’s (2014) finding using postural control measures. Future studies should confirm the association between postural control and kinesthetic experience by simultaneously collecting third-person and phenomenological data (in preparation).

In addition to the physiological measures, our phenomenological results show that participants perceived temporal fluctuations during their empathic experiences. One aspect that temporally fluctuated was bodily sensations (visceral and muscular). At the beginning of the video, participants experienced a gradual increase in the intensity of bodily sensations; this intensity then peaked as the anticipated accident occurred; finally, after the accident, they felt a physical relaxation in their bodies. The critical point of this discussion concerns the quantitative methods used to analyze postural control and physiological responses in studies of social emotions. Traditionally, these studies consider sensorimotor and somatic body reactions as a linear process that is averaged throughout a time analysis (e.g., Gea et al., 2014; Hagenaars et al., 2014; Brandão et al., 2016). This approach precludes revealing the temporal dynamics of body movements and the physiological responses, as observed in our phenomenological results. Therefore, we call for future studies to implement non-linear analysis methods to detect the motor and physiological temporal dynamics (e.g., Van Emmerik et al., 2016; Jauniaux et al., 2020).

Traditionally, social neuroscience has identified two types of empathic responses to observing someone in discomfort: empathic concern and personal distress (e.g., Batson et al., 1987; Eisenberg and Eggum, 2009; Jordan et al., 2016). Empathic concern, also called sympathy, is an other-oriented emotional response congruent with what the other is perceived to be experiencing. By contrast, personal distress is a self-oriented emotional response focused on one’s own sensations (Okun et al., 2000). Empathic concern leads individuals to focus on the other’s well-being and results in prosocial behaviors, whereas personal distress mobilizes behaviors to reduce one’s own suffering (Singer and Lamm, 2009). We found similar evidence but from a phenomenological perspective. In self-centered individuals, attention to the other generated intense feelings of discomfort and the wish to avoid the situation; conversely, other-centered participants focused attention on what happens to the sportspersons and the possible consequences of the accidents, which motivated prosocial behavior. Although the concepts of empathic concern and personal distress appear similar to what we observed phenomenologically, they provide different perspectives on the same phenomenon. The first provides a naturalized comprehension of empathy for pain (Gallagher, 2011; Olivares et al., 2015), while the second offers a holistic perspective, integrating a repertoire of sensitive, affective, somatic, sensorimotor, and cognitive experiences that change temporally (as discussed further below). Finally, when unraveling the concept of compassion in this study, our phenomenological data support that participants with other-centered empathic style showed a compassionate attitude toward the sportspersons, in the sense that those participants directed their attention toward others and manifested a prosocial kinesthetic motivation to help the suffering person. Nevertheless, sensations and feelings such as warmth, care, and benevolence, generally associated with compassion in the contemplative neuroscience field (Singer and Klimecki, 2014), did not emerge in the interviews. Therefore, our phenomenological results are in accordance with findings of compassion experience without pleasant or positive emotions in participants without previous meditation practice (Condon and Barrett, 2013).

### Empathic experience for pain and the enactive dimensions of embodiment

This subsection further discusses the relationship between empathy and other mental and biological processes, specifically addressing how empathy relates to interoceptive, affective, and sensorimotor processes, from the first-person perspective, and under an *enactive* cognitive science framework. To contextualize, the enactive approach of Varela and colleagues has been essential to incorporating phenomenology into cognitive science (Varela et al., 1991; Varela, 1996; Thompson, 2007), and a clinical perspective of the human being based on physical, subjective, and environmental attributes (De Jaegher, 2013; De Haan, 2020; Martínez-Pernía, 2020; Martínez-Pernía et al., 2021).

It is also important to clarify that the enactive approach is a specific research program within a much larger set of embodied approaches (Thompson, 2007; Di Paolo et al., 2017). These approaches can be broadly divided into two incompatible sets of views. One is embodied functionalism (Di Paolo et al., 2017), also called “the body snatchers” (Gallagher, 2015), or what Clark (1999) refers to by simple embodiment. While extending the focus of research from solely the brain to also include the effect of the body and the environment on mental processing, nonetheless retain the view that cognition and affect essentially consist of the manipulation of representations and performance of mental functions inside the head. In contrast, the other trend, which Thompson (2007) calls embodied dynamicism, and Clark (1999) radical embodiment, rejects the appeal to both representations and functional properties/states, and sees the body, including its brain, as a dynamical, self-organizing, emergent system in interaction with the environment, giving rise to cognition and affect. The enactive approach fits within this latter camp and aims to build bridges between dynamical accounts of the brain-body-environment interaction and the phenomenology of lived experience. Our study contributes to this, by experimentally enhancing our knowledge of the phenomenology of empathy, and suggesting an extension of the enactive theory concerning social experience, as we will see in more detail below. As a final clarification, we would also like to mention that there are three, different accounts that are usually called “enactive” (Ward et al., 2017). One is radical enactivism (Hutto and Myin, 2013), the other is sensorimotor enactivism (O’Regan and Noë, 2001), and finally, there is the so-called autopoietic enactivism of Varela et al. (1991). Although all reject the representationalist, computationalist, functionalist understanding of cognition and affect, and share the emphasis on adaptive, sensorimotor interaction between agent and environment, they also differ in many respects and we want to make clear that we are focusing on the latter, Varelian, autopoietic variant^4^.

In particular, we will focus on the *three dimensions of embodiment* (Thompson and Varela, 2001; Varela and Thompson, 2003), premised on the claim that consciousness-relevant brain activity should be understood in the context of the three cycles of operation in which the brain participates within the living organism. The first cycle is the process of *organismic regulation* within the human body; the second is the *sensorimotor coupling* of the conscious agent with its environment; and the third is the *intersubjective interaction* between two or more conscious agents. Hence, consciousness—including empathic experience—arises through not just the brain but its dynamic coupling with the living body in a world including other conscious organisms (Thompson and Varela, 2001; Varela and Thompson, 2003).

Before discussing the place of empathy in Varela and Thompson’s account, and how it relates to our phenomenological data, we need to give more details about the three cycles. First, in the *organismic regulation* cycle, the brain interacts with the rest of the body, especially through interoceptive and autonomic pathways, to secure the homeostatic balance needed for preserving life and health. Importantly, the experiential correlates of this activity are bodily sensations and feelings including pains, tickles, hunger, thirst, and muscular tension; emotional states such as fear, distress, and happiness; and a basic affective and embodied sense of selfhood (Damasio, 1999, 2018; Craig, 2002, 2015; Barrett, 2017; Seth and Tsakiris, 2018; Carvalho and Damasio, 2021). Second, the *sensorimotor coupling* cycle between the agent and its environment has perceptual and kinesthetic experiential correlates (Thompson and Varela, 2001; Varela and Thompson, 2003). Its main tenet is the reciprocal relationship between movement and perception in the unfolding of experience: what (and how) we perceive is a function of how we move (or would potentially move), and how we move is a function of what we perceive (Varela et al., 1991; O’Regan and Noë, 2001; Noë, 2006; Thompson, 2007). Third, the *intersubjective interaction* cycle between two or more subjects is the domain of psychology and neuroscience studies of social cognition and emotions. Notably, Varela and Thompson (2003: 14) state that the experiential dimension of “social cognition is empathy, in the broad sense of the affectively mediated experience of self and other.” From the enactive perspective, then, empathy is central to situations in which we think and feel in relation to others, as those capacities presuppose that we experience others as *subjects* of experience (not just physical entities with complex behaviors), that could also have thoughts and feelings about us.

Crucially, Thompson and Varela (2001: 424) state that both the “affective state and sensorimotor coupling plays a huge role in social cognition, especially in apes and humans,” alluding to the idea that the first two cycles play a key role in the third (intersubjective) one. However, somewhat contradicting their emphasis on embodiment and consciousness, their phenomenological description is very limited; they mainly cite evidence that cerebral structures important in social cognition are also key in emotion (e.g., the amygdala), and the “mirror neurons” studies mentioned earlier. This is related to the fact that social cognition research has frequently employed third-person methodologies (Gallagher, 2012), such as functional magnetic resonance imaging, electroencephalogram, physiological activity, and motor control (Neumann et al., 2015). Although these methodologies are undoubtedly very valuable, our research has two clear advantages in uncovering the phenomenology of an empathic experience: it uses an easily replicable experimental setting previously employed in affective neuroscience (e.g., Azevedo et al., 2005; Hagenaars et al., 2012, 2014), and it applies a well-defined methodology to assess participants’ first-person experience. We, thus, consider that our results contribute to experimentally filling the phenomenological gap within the enactive approach and also in the field of social cognition and social emotions more generally. Although the enactive approach has been extended in both phenomenological and dynamical terms to account for intersubjectivity [De Jaegher and Di Paolo, 2007; Fuchs and de Jaegher, 2009; see also the review by Lindblom (2020)], and the philosophical phenomenology of empathy has been discussed within enactivism (Thompson, 2001, 2007), the phenomenological gap has not been filled empirically, nor specifically in relation to empathy for pain in non-interactive situations^5^, and in that sense our study represents a contribution to the enactive approach.

Very importantly, our study also contributes to a more complete theoretical understanding of intersubjectivity within the enactive framework itself, which has been developed with an exclusive focus on interactive situations (Thompson and Varela, 2001; De Jaegher and Di Paolo, 2007; De Jaegher and Froese, 2009; Fuchs and de Jaegher, 2009; Froese et al., 2014; De Jaegher, 2015; De Jaegher et al., 2017), thus, neglecting non-interactive social experiences like dreaming about someone, imagining dancing with a friend, or seeing the video-recording of someone having a painful experience (as in our case). In other words, while the enactive approach to intersubjectivity until now has focused exclusively on understanding the dynamics and phenomenology of actual interactions between two or more agents, there is a whole set of social experiences that are left outside, in which there is no actual interaction between the agent and the subjects that appear in her experience. Based on our study, we would like to offer a sketch of how the enactive theory may be extended to account for these non-interactive social experiences.

If enactivism is right that both autonomy and its concomitant twofold process of sense-making and identity are the basis of mind (Varela, 1984; Thompson, 2007, 2011), we can say there is a non-interactive form of empathic sense-making and identity, grounded on the actual autonomous activity of the empathizer in their environment (which could exclude the physical presence of other subjects); this would enact an experience in which other experiential subjects are also present, probably due to a previous history of intersubjective interactions (Fuchs, 2017). A reasonable hypothesis, then, is that previous intersubjective interactions may trigger changes in the autonomous organization underlying both organismic regulation and sensorimotor coupling (comprising the central nervous system but plausibly also global properties of bodily configuration such as musculature and homeostatic processes), enabling a non-interactive form of social identity and sense-making to be enacted through the operationally closed activity of the organismic and sensorimotor cycles alone. As mentioned, this hypothesis and the future studies we expect will be motivated by it, represent an extension of the current enactive approach to social experience because it enlarges the scope of the theory to account for non-interactive situations in which, nonetheless, other subjects experientially appear to an agent.

Concerning our findings on the self-centered and other-centered empathic experiential structures, we hypothesize that the operation of the first two cycles (i.e., homeostatic and sensorimotor), with their experiential correlates, may determine or influence the extent to which the individual focuses concern on herself or the other in intersubjective experiences. This is compatible with prior findings that when confronted with someone in need, a subject’s degree of experienced empathic concern and personal distress is correlated with her/his ability to emotionally self-regulate (Eisenberg and Spinrad, 2014), which, in turn, is tightly related to interoceptive/homeostatic regulation (Craig, 2015; Barrett, 2017). Our results also indicate that the kinesthetic dimension marks an important difference between self-centered and other-centered experiences. For some participants, kinesthetic sensations and motivations were expressive of more self-centered concern and the will to avoid or escape the situation (self-centered structure); for others, kinesthetic experiences were more directed to the sportspersons, often with the intention of helping (other-centered structure). This suggests that the difference between experiencing personal distress and experiencing empathy may not be entirely explained by “cognitive focus” (Maibom, 2017: 3) and that the kinesthetic component, which is a key phenomenological element of the second cycle, could also play a key role that has been virtually disregarded by researchers. Our results also showed that the empathic experience of seeing others in pain is neither an exclusively cognitive process of mentalizing, i.e., *cognitive empathy* (Spaulding, 2017) usually explained in terms of TT, ST, or a mixture of both, nor an exhaustively affective-cognitive process in which an affective emotional quality is accompanied by a certain type of cognitive evaluation and focus, i.e., *affective empathy* (Maibom, 2017). Instead, our study indicated that empathic experience is a far more complex process in which not just affect (e.g., emotions and moods) and cognition (e.g., thoughts and attention) participate, but also a bodily resonance comprising visceral and muscular sensations, and also a key kinesthetic component related to the experience of moving or wanting to move in a certain way. This could have important implications for the future study of affective empathy, as our investigation suggests that considering the somatic, sensorimotor, and temporal phenomenological dimensions could shed important light on the affective and cognitive elements themselves, while also providing a more complete picture of the whole experiential process taking place.

## Conclusion

According to Neumann et al. (2015), empathy has been evaluated through self-report, behavioral and neuroscientific measures, but first-person methods based on the phenomenological experience have been neglected (Gallagher, 2011; Fuchs, 2013), leaving little understanding of empathy from the observer’s subjective experience. Hence, our main aim was to contribute, empirically, to enlarge our little scientific knowledge of the empathic experience of pain by offering the first investigation of empathic experience through an experimental phenomenological method. In this way, our study significantly contributes to filling the “phenomenological gap” in the empirical study of empathy by employing a clearly defined procedure by which the lived experience of empathizers is collected and analyzed. Overall, our study revealed two experiential structures—self-centered empathy and other-centered empathy—that differ in how the subjects show concern for people suffering pain. In particular, our results reveal that understanding of the other person occurs through bodily resonance and involves an integrated multiplicity of bodily sensations, negative emotions, motivations, and thoughts that fluctuate through time. Furthermore, we showed how our findings may contribute to advancing our understanding of empathy in social neuroscience, and how they may enhance the enactive approach to social experience and intersubjectivity. We also suggested how our study can enhance our concepts of empathy by highlighting that beyond the dichotomies of cognitive/affective empathy, empathic concern/personal distress; empathic experience is a rich and complex process with gradualities along several dimensions, in which the lived body plays an essential but most often neglected role.

However, our study also has some limitations. First, we instructed participants to remain still while watching the videos. This certainly narrowed to a great extent what we found concerning kinesthetic sensations and the sensorimotor cycle in general. Future studies should encourage full expression of movement and bodily postures, such that the sensorimotor cycle is not artificially restricted even in non-interactive situations. A second limitation is that our study only collected phenomenological data, while a more integral understanding of the target phenomenon probably requires simultaneously collecting physiological, neural, and phenomenological measures; and finding out the relations between them, as in neurophenomenology (Varela, 1996).

Finally, future studies should test the hypotheses and suggestions that stem from our study concerning empathy research in social neuroscience, such as whether interoceptive and kinesthetic sensations in the empathic experience are more correlated with lower-level or higher-level processing in the brain; and the implications for the enactive approach, such as the specific ways in which intersubjective interactions shape the structures supporting the homeostatic and sensorimotor cycles and the role these cycles may then play in non-interactive empathic experiences.

## Data availability statement

The original contributions presented in this study are included in https://osf.io/fd7vt/, further inquiries can be directed to the corresponding author.

## Ethics statement

The studies involving human participants were reviewed and approved by all the participants signed informed consent. The study procedure was according to the Declaration of Helsinki principles. It was approved by the “Scientific Ethics Committee of the Servicio de Salud Metropolitano Oriente” and the “Research in Humans being Ethics Committee of the Medicine Faculty, Universidad de Chile.” The patients/participants provided their written informed consent to participate in this study.

## Author contributions

DM-P conceived the study, constructed and validated the videos, and implemented the phenomenological interviews. DM-P, AT, and KB did the phenomenological analysis. AT and KB analyzed the quantitative data. CB and CA-V wrote the codebook. DM-P, IC, JC, VC, VM, and MV wrote the first version of the manuscript. DM-P, IC, AT, KB, CB, and CA-V wrote the final version of the manuscript. AU-O and DH constructively reviewed the manuscript. All authors contributed to the article and approved the submitted version.

## Footnotes

1 The third-person view refers to the objective procedures to study cognition through technological devices (e.g., fMRI and EEG). The first-person view refers to the meaning reports of the participant’s lived experience (e.g., self-report and phenomenological experience). The second-person view is the implementation of interview procedures applied by the researcher to collect the participant’s experience. In this study, we implemented a phenomenological interview (second-person view) to collect participants’ phenomenological experience (first-person view).

2
https://osf.io/fd7vt/

3 This study also collected physiological and behavioral data through third-person methods such as force platform, electrocardiogram, and galvanic skin response. However, this paper focuses only on the phenomenological data.

4 Hereafter, by “enactive approach” and “enactivism” we will refer to the autopoietic, Varelian, original version of enactivism.

5 By “non-interactive situations” we mean instances of social cognition and emotion where the experimental subject is not interacting with other subjects (in our experimental setting the sportspersons seen in the TV screen) whose painful experiences are, nonetheless, an integral part of the experimental subject’s empathic experience.
